# The Efficacy of Intensive Statin Therapy in Acute Ischemic Stroke Following Intravenous Thrombolysis: The CASE II Study

**DOI:** 10.1111/cns.70186

**Published:** 2025-01-13

**Authors:** Fujian Chen, Huan Zhou, Tingxia Zhang, Liangxue Wang, Hongfang Chen, Jin Hu, Guomin Xie, Shenqiang Yan, Min Lou, CASE‐II Investigators

**Affiliations:** ^1^ Department of Neurology People's Hospital of Anji Huzhou China; ^2^ Department of Neurology The Second Affiliated Hospital of Zhejiang University, School of Medicine Hangzhou China; ^3^ Department of Neurology Affiliated Jinhua Hospital, Zhejiang University School of Medicine Jinhua China; ^4^ Department of Neurology The First Affiliated Hospital of Jiaxing University Jiaxing China; ^5^ Department of Neurology Ningbo Medical Center Li Huili Hospital Ningbo China

**Keywords:** ischemic stroke, low‐density lipoprotein, real‐world study, statin, thrombolysis

## Abstract

**Aims:**

This study aimed to investigate the efficacy of early intensive statin therapy following intravenous thrombolysis (IVT) in patients with acute ischemic stroke (AIS).

**Methods:**

AIS patients who received IVT and statin therapy were included from multicenter registry databases. The primary endpoint was functional independence, defined by a modified Rankin Scale (mRS) score of 0–2 at 90 days. Propensity score matching (PSM) analyses were employed.

**Results:**

A total of 21,349 patients were included in this study, with a mean age of 68.5 ± 12.6 years, of whom 13,578 (63.6%) were male. The baseline NIHSS score was 4 (IQR 2–8). A total of 9532 patients (44.6%) received intensive statin therapy. In the PSM analysis, the proportion of patients with mRS scores of 0–2 was significantly higher in the intensive statin therapy group (OR = 1.095, 95% CI 1.022–1.173, *p* = 0.010). Statin type modified the effect of intensive statin therapy on functional independence (*p*‐value for interaction = 0.030). Treatment effects favoring the intensive approach were observed in patients receiving atorvastatin (OR = 1.134, 95% CI 1.051–1.224, *p* = 0.001).

**Conclusion:**

Early intensive statin therapy following IVT leads to a significant but modest improvement in neurological outcomes, particularly in patients treated with atorvastatin as part of the intensive regimen.

## Graphical abstract

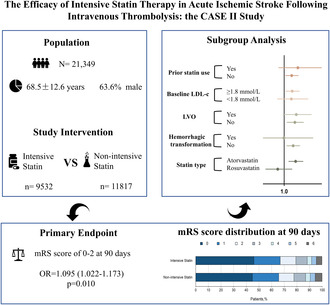

## 1 Introduction

Intravenous thrombolysis (IVT) is the most effective pharmacological treatment for acute ischemic stroke (AIS) [1]. Additionally, early management strategies such as blood pressure control, blood glucose regulation, and lipid‐lowering therapy are essential in treating ischemic stroke [2, 3]. Statins, as the cornerstone of lipid‐lowering drugs, are known to reduce the incidence of future cardiovascular and cerebrovascular events in patients with ischemic stroke or transient ischemic attack, particularly with intensive statin use [4, 5]. Moreover, previous studies have shown that in addition to reducing the risk of first and recurrent strokes, statins may also improve outcomes after AIS, demonstrating neuroprotective effects [6, 7].

Some studies have found that early statin therapy after IVT can improve short‐ and long‐term neurological outcomes in stroke patients [8, 9, 10]. However, it remains unclear whether intensive statin therapy offers greater benefits in patients undergoing IVT. Currently, the Intensive Statin Plus Intravenous rt‐PA in Acute Ischemic Stroke (INSPIRE) trial is the only randomized controlled study evaluating the efficacy of early intensive statin therapy after IVT. The results showed no significant differences in neurological outcomes and safety between the treatment and control groups [11]. However, the INSPIRE trial used only an intensive regimen of rosuvastatin and did not identify subgroups of patients who might benefit from intensive statin therapy [11].

Thus, this study aims to analyze detailed statin regimens in patients receiving statin therapy after IVT, using data from a multicenter, large‐sample database to explore the effectiveness of intensive statin therapy on neurological outcomes based on real‐world data.

## 2 Methods

### 2.1 Subjects

The Computer‐based Online Database of Acute Stroke Patients for Stroke Management Quality Evaluation (CASE‐II, NCT04487340) is a prospective multicenter stroke registry in China. The primary aim of this project is to establish an online database of AIS patients to assess the quality of stroke management. In the current study, we collected patients who met the following inclusion criteria from January 1, 2017, to May 31, 2023: (1) aged over 18 years; (2) had a clinical diagnosis of AIS; (3) received IVT with recombinant tissue plasminogen activator (rt‐PA); (4) received statin therapy during hospitalization. We excluded patients who: (1) the length of hospitalization ≤ 2 days; (2) received endovascular treatment during hospitalization; (3) had a pre‐stroke modified Rankin Scale (mRS) score ≥ 2; (4) had incomplete records related to statin therapy, such as medication type, specific dosage, and duration of use; (5) experienced changes in statin type or dosage during hospitalization within 1 week; (6) were lost to follow‐up at 90 days. This study followed the STROBE guidelines.

### 2.2 Ethics Statement

This study was approved by the human ethics committees of the Second Affiliated Hospital of Zhejiang University and each participating subcenter (2016.11, yan2016‐064). All clinical investigations adhered to the principles outlined in the Declaration of Helsinki. Written informed consent was obtained from all participants to confirm their agreement to undergo IVT.

### 2.3 Data Collection

Clinical data were registered by well‐trained personnel according to standard protocols, and the quality of the database was verified by a professional committee. We retrieved patient‐level data, including demographics (age, sex), medical history (hypertension, atrial fibrillation, diabetes mellitus, prior ischemic stroke, and smoking), prior antiplatelet and statin use, baseline National Institutes of Health Stroke Scale (NIHSS) score at admission before IVT, baseline serum low‐density lipoprotein cholesterol (LDL‐C) measured on the first morning after admission (potentially after the initiation of statin therapy following IVT), presence of intracranial large vessel occlusion, hemorrhagic transformation (HT) after IVT, details of the statin therapy strategy, and mRS score at 90 days. Patients initiated statin therapy within 48 h of stroke onset and continued for at least 1 week or throughout the entire hospitalization period.

The Guideline on the Management of Blood Cholesterol considers intensive statin therapy to typically lower LDL‐C levels by ≥ 50%, using either atorvastatin 40–80 mg or rosuvastatin 20–40 mg [12]. Therefore, in this study, the intensive statin group was defined as patients receiving atorvastatin ≥ 40 mg/day or rosuvastatin ≥ 20 mg/day, whereas all other regimens were classified as the non‐intensive statin group.

### 2.4 Imaging Analysis and Outcome Assessment

Pre‐thrombolytic imaging assessment of intracranial large vessel occlusion encompasses occlusions in the middle cerebral artery, internal carotid artery, anterior cerebral artery, basilar artery, vertebral artery, and posterior cerebral artery. HT following IVT was evaluated by an imaging core laboratory in accordance with ECASS II criteria [13]. The primary outcome of this study was functional independence, which was defined as mRS score of 0–2 at 90 days. The secondary outcomes consisted of excellent functional outcome (mRS 0–1) at 90 days and mRS distribution at 90 days. Outcome data at 90 days were gathered through standardized telephone interviews with patients or their relatives, conducted by certified external clinical evaluators using standardized forms. All interviews were recorded and traceable.

### 2.5 Statistical Analysis

Participants in the study were categorized into two groups: the intensive statin group and the non‐intensive statin group. Data were expressed as mean ± standard deviation (SD), median (interquartile range [IQR]), or count and percentage (No. [%]). For continuous variables, comparisons between groups were made using either the independent samples *t*‐test or the Mann–Whitney *U* test, depending on the data distribution, which was assessed by the Kolmogorov–Smirnov test for normality. Categorical variables were compared using the Chi‐square test or Fisher's exact test, as appropriate. Propensity score matching (PSM) was conducted using a 1:1 nearest‐neighbor matching algorithm with a caliper width of 0.1 to balance baseline characteristics between the groups. Multiple imputation methods were applied to address missing baseline data. Statistical analyzes were performed using SPSS version 26.0, with a significance threshold set at *p* < 0.05.

## 3 Results

**FIGURE 1:**
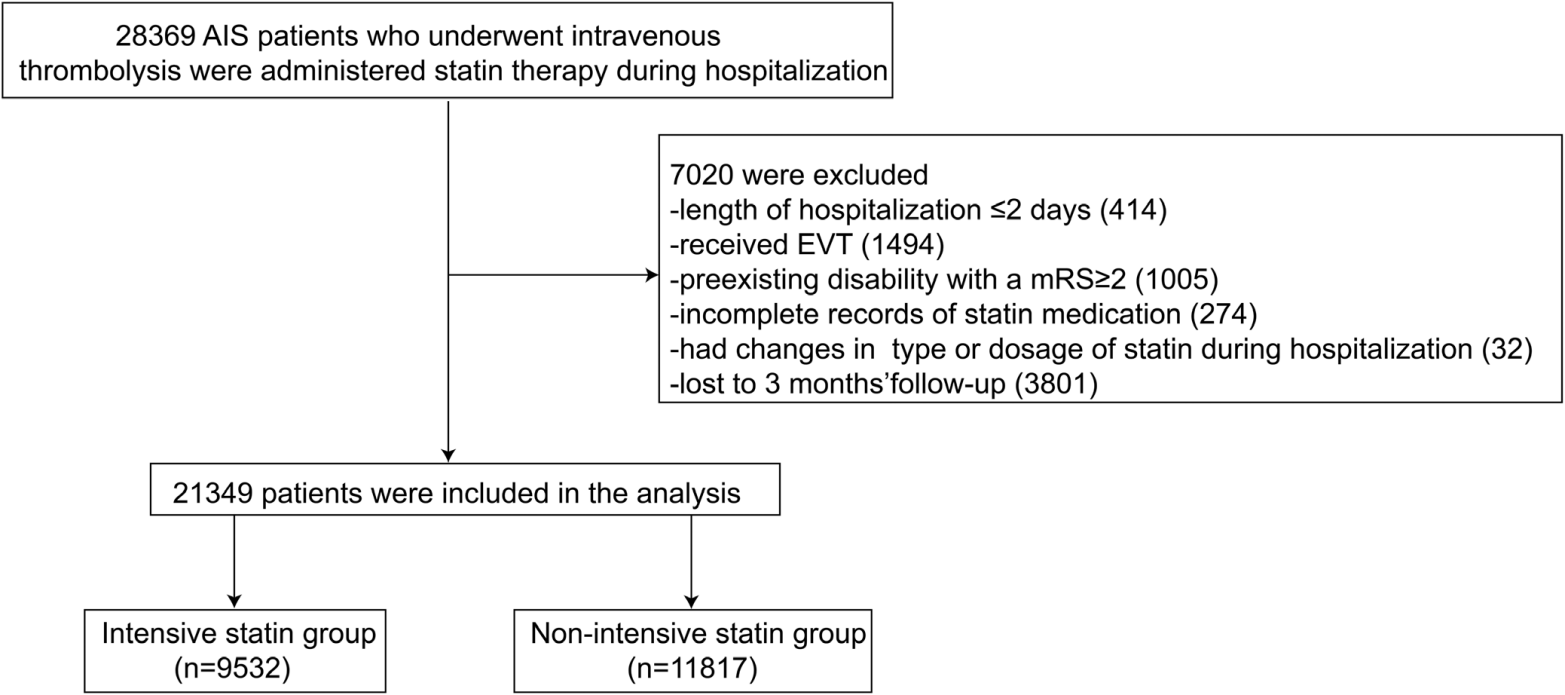
Flowchart of enrollment and treatment. AIS, acute ischemic stroke; EVT, endovascular treatment; mRS, modified Rankin Scale. Intensive statin group: Atorvastatin ≥ 40 mg/day or rosuvastatin ≥ 20 mg/day. Non‐intensive statin group: Atorvastatin < 40 mg/day or rosuvastatin < 20 mg/day. Statin therapy was initiated within 48 h of stroke onset and continued for at least 1 week or throughout hospitalization.

As shown in the Figure 1, a total of 28,369 AIS patients who underwent IVT were administered statin therapy during hospitalization in the study period, of which 7020 patients were excluded for the following reasons: 414 patients' length of hospitalization ≤ 2 days; 1494 patients underwent endovascular treatment; 1005 patients had a preexisting mRS score ≥ 2; 274 patients had incomplete statin‐related records; 32 patients had a change in statin type or dosage during hospitalization; and 3801 patients were lost to follow‐up at 3 months. Ultimately, a total of 21,349 patients were included in the study. The average age was 68.5 ± 12.6 years, with 13,578 (63.6%) male patients. The baseline NIHSS score was 4 (IQR 2–8), and the baseline LDL‐C level was 2.65 ± 0.87 mmol/L. A total of 9532 patients (44.6%) received intensive statin therapy.

### 3.1 Baseline Characteristics Before and After PSM

Table 1 compares the characteristics of patients who received intensive statin therapy with those who did not. The intensive statin group was younger, had a lower incidence of atrial fibrillation, a relatively lower baseline NIHSS score, and a lower occurrence of HT after IVT. There were no differences between the two groups in terms of prior statin use, baseline LDL‐C levels, and the type of statin used. After PSM (Figure S1), we matched a total of 9532 pairs of patients, achieving a statistical balance in baseline variables between the two groups (Table 1).

**TABLE 1:** Unmatched and propensity‐matched analysis of intensive statin therapy effects on acute ischemic stroke patients receiving intravenous thrombolysis.

Variables	Unmatched		ASD	Propensity‐matched		ASD
	Intensive statin					
	Yes (*n* = 9532)	No (*n* = 11,817)		Yes (*n* = 9532)	No (*n* = 9532)	
Age (year)	67.7 ± 12.5	69.1 ± 12.6	**0.108**	67.7 ± 12.5	68.6 ± 12.4	0.067
Male, %	6180 (64.8)	7398 (62.6)	0.047	6180 (64.8)	6032 (63.3)	0.033
Medical history						
Hypertension, %	6137 (64.4)	7525 (63.7)	0.015	6137 (64.4)	6042 (63.4)	0.021
Atrial fibrillation, %	1371 (14.4)	2229 (18.9)	**0.128**	1371 (14.4)	1375 (14.4)	0.001
Diabetes mellitus, %	1726 (18.1)	1998(16.9)	0.031	1726 (18.1)	1634 (17.1)	0.025
Prior ischemic stroke, %	1118 (11.7)	1434 (12.1)	0.013	1118 (11.7)	1133 (11.9)	0.005
Smoking, %	2977 (31.2)	3459 (29.3)	0.042	2977 (31.2)	2836 (29.8)	0.032
Prior antiplatelet use, %	1148 (12.0)	1632 (13.8)	0.054	1148 (12.0)	1256 (13.2)	0.035
Prior statin use, %	871 (9.1)	1142 (9.7)	0.018	871 (9.1)	878 (9.2)	0.003
Baseline NIHSS score	4 (2–8)	4 (2–9)	**0.103**	4 (2–8)	4 (2–8)	0.017
Baseline LDL‐c (mmol/L)	2.68 ± 0.87	2.63 ± 0.86	0.051	2.68 ± 0.87	2.65 ± 0.85	0.032
Intracranial LVO, %	1561/8983 (17.4)	1812/11146 (16.3)	0.030	1561/8983 (17.4)	1356/9012 (15.0)	0.062
Hemorrhagic transformation	358 (3.8)	692 (5.9)	**0.110**	358 (3.8)	348 (3.7)	0.006
In‐hospital statin type			0.082			0.084
Atorvastatin	8047 (84.4)	9625 (81.5)		8047 (84.4)	7758 (81.4)	
Rosuvastatin	1485 (15.6)	2192 (18.5)		1485 (15.6)	1774 (18.6)	

*Note:* Bold values indicate an ASD > 0.1, which reflects a meaningful imbalance between groups.

Abbreviations: ASD, absolute standardized difference; LDL‐c, low‐density lipoprotein cholesterol; LVO, large vessel occlusion; NIHSS, National Institute of Health Stroke Scale.

### 3.2 Primary and Secondary Outcomes

In the PSM analysis, the proportion of patients with mRS scores of 0–2 was significantly higher in the intensive statin therapy group (OR = 1.095, 95% CI 1.022–1.173, *p* = 0.010). There was also a trend toward a higher proportion of patients with mRS scores of 0–1 in the intensive statin therapy group (OR = 1.054, 95% CI 0.993–1.119, *p* = 0.086). Additionally, intensive statin therapy was associated with lower mRS scores at 3 months (OR = 0.940, 95% CI 0.892–0.990, *p* = 0.020) (Table 2, Figure S2).

**TABLE 2:** Functional outcomes of intensive statin therapy versus non‐intensive statin therapy in propensity‐matched acute ischemic stroke patients receiving intravenous thrombolysis.

Functional outcome	Intensive statin		OR (95% CI)	*p*
	Yes (*n* = 9532)	No (*n* = 9532)		
Primary outcome				
mRS score of 0–2 at 90 days, %	7556 (79.3)	7410 (77.7)	**1.095 (1.022–1.173)**	**0.010**
Secondary outcomes				
mRS score of 0–1 at 90 days, %	6373 (66.9)	6261 (65.7)	1.054 (0.993–1.119)	0.086
mRS score distribution at 90 days			**0.940 (0.892–0.990)**	**0.020**
0, %	4389 (46.0)	4275 (44.8)		
1, %	1984 (20.8)	1986 (20.8)		
2, %	1183 (12.4)	1149 (12.1)		
3, %	860 (9.0)	877 (9.2)		
4, %	335 (3.5)	357 (3.7)		
5, %	357 (3.7)	375 (3.9)		
6, %	424 (4.4)	513 (5.4)		

*Note:* Bold values indicate statistical significance (*p* < 0.05).

Abbreviations: CI, confidence interval; mRS, modified Rankin Scale; OR, odds ratio.

In the subgroup analyses with mRS scores of 0–2 as the end point, some interesting findings emerged. We found that treatment effects favoring intensive statin therapy were more evident in patients without prior statin use (OR = 1.088, 95% CI 1.011–1.170, *p* = 0.024), with baseline LDL‐C ≥ 1.8 mmol/L (OR = 1.095, 95% CI 1.015–1.182, *p* = 0.019), without intracranial large vessel occlusion (OR = 1.126, 95% CI 1.032–1.229, *p* = 0.007), without HT after IVT (OR = 1.105, 95% CI 1.028–1.187, *p* = 0.006), those who received atorvastatin for intensive therapy (OR = 1.134, 95% CI 1.051–1.224, *p* = 0.001), and those with a stroke etiology of non‐cardioembolism (OR = 1.105, 95% CI 1.021–1.195, *p* = 0.013). It is noteworthy that the distinction between atorvastatin and rosuvastatin may be definitive and not merely because of sample size differences (*p*‐value for interaction = 0.030) (Table 3).

**TABLE 3:** ORs and 95% CIs for modified Rankin Scale score ≤ 2 across subgroups in propensity‐matched acute ischemic stroke patients receiving intravenous thrombolysis.

Subgroup	Intensive statin		OR (95% CI)	*p*	*p*_het_
	Yes	No			
	No. of patients				
Prior statin use					
Yes	871	878	1.161 (0.934–1.442)	0.179	0.580
No	8661	8654	**1.088 (1.011–1.170)**	**0.024**	
Baseline LDL‐c					
≥ 1.8 mmol/L	8096	8037	**1.095 (1.015–1.182)**	**0.019**	0.920
< 1.8 mmol/L	1436	1495	1.085 (0.918–1.283)	0.340	
Intracranial LVO					
Yes	1561	1356	1.142 (0.986–1.322)	0.076	0.876
No	7422	7656	**1.126 (1.032–1.229)**	**0.007**	
Hemorrhagic transformation					
Yes	358	348	0.997 (0.742–1.340)	0.986	0.510
No	9174	9184	**1.105 (1.028–1.187)**	**0.006**	
In‐hospital statin type					
Atorvastatin	8047	7758	**1.134 (1.051–1.224)**	**0.001**	**0.030**
Rosuvastatin	1485	1774	0.924 (0.781–1.094)	0.358	
Stroke etiology					
Large‐artery atherosclerosis	2773	2805	1.100 (0.977–1.238)	0.114	0.959
Cardioembolism	1404	1440	1.051 (0.903–1.223)	0.522	
Small‐artery occlusion	2633	2901	1.151 (0.978–1.306)	0.091	
Stroke of other determined cause	36	61	0.528 (0.179–1.558)	0.243	
Stroke of undetermined cause	2686	2325	1.130 (0.975–1.311)	0.104	

*Note:* Bold values indicate statistical significance (*p* < 0.05).

Abbreviations: CI, confidence interval; LDL‐c, low‐density lipoprotein cholesterol; LVO, large vessel occlusion; OR, odds ratio; *p*_het_, heterogeneity *p* value.

## 4 Discussion

This study investigates the application and efficacy of early intensive statin therapy following IVT, using data from a large multicenter database. In real‐world practice, the early initiation of intensive statin therapy after AIS is relatively well‐accepted among neurologists in China, with nearly 50% of AIS patients who underwent IVT receiving early intensive statin therapy. Our findings show that early intensive statin therapy after IVT is associated with better neurological outcomes compared to non‐intensive statin therapy. Moreover, the beneficial effects of intensive statin therapy may be primarily driven by atorvastatin rather than rosuvastatin.

Statins not only reduce cholesterol and low‐density lipoprotein levels but also exert neuroprotective effects, such as endothelial protection, improved cerebral blood flow, and anti‐inflammatory properties [14, 15]. A previous meta‐analysis demonstrated that statin use during ischemic stroke significantly improved neurological outcomes at 3 months, reducing the risks of death, disability, and stroke recurrence [16]. Although research specifically focusing on IVT patients is limited, statin therapy during the acute phase has shown favorable impacts on clinical outcomes [8, 9, 10, 17]. Current guidelines recommend continuing statin therapy for patients already on statins prior to stroke and initiating statin therapy early in eligible patients [1]. Further exploration of intensive statin therapy in AIS patients, including those treated with IVT, is clinically relevant.

A recent meta‐analysis indicated that, compared to non‐intensive statin therapy, intensive statin pretreatment significantly improved coronary microvascular function after percutaneous coronary intervention (PCI) [18]. The INSPIRE trial evaluated the effect of early combined therapy with 20 and 5 mg of rosuvastatin on functional outcomes after IVT, but the results were negative [11]. In our study, the intensive statin regimen was not limited to 20 mg rosuvastatin but adhered to guideline definitions, which included atorvastatin 40–80 mg or rosuvastatin 20–40 mg [12]. Our findings suggest that early intensive statin therapy after IVT can significantly improve neurological outcomes at 90 days, although the improvement is modest (79.3% versus 77.7%). In the INSPIRE trial, the intensive statin group and the control group showed mRS 0–2 rates of 81.9% versus 79.4%, respectively [11]. Clearly, such modest differences may not be detectable in studies with smaller sample sizes of only a few hundred cases.

Furthermore, we conducted additional subgroup analyses to identify patient populations that might benefit more from intensive statin therapy, leveraging the large sample size of our study. The most significant subgroup analysis focused on the type of statin used. We found that patients receiving intensive atorvastatin showed improved neurological outcomes compared to those on non‐intensive atorvastatin; however, no such difference was observed with rosuvastatin. Although some studies suggest that rosuvastatin may be superior to atorvastatin in lowering LDL cholesterol levels [19, 20], this could limit the potential for improved outcomes following intensive dosing. Additionally, evidence indicates that high‐dose atorvastatin may be safer than rosuvastatin, which could mitigate some benefits [21, 22]. A single extensive dose of lipophilic atorvastatin prior to primary PCI demonstrated better improvement in microvascular myocardial perfusion compared to hydrophilic rosuvastatin [23, 24]. This may also explain the negative results of the INSPIRE trial, aside from sample size considerations.

Although no significant interactions were found in other subgroup analyses, some findings could still be rationalized. In patients with baseline LDL‐C ≤ 1.8 mmol/L, the benefits of intensive statin therapy seemed less pronounced, possibly because prior statin use might have already “pre‐spent” the potential benefits, a concept known as the “saturation effect” [25]. This is consistent with the findings of Cappellari et al., who noted that statin treatment initiated before stroke and continued in the acute phase may not improve short‐ and long‐term outcomes after IVT [8]. Consequently, the “saturation effect” could explain why patients without prior statin use experienced greater benefits from intensive therapy. Those who had not received statins before IVT demonstrated greater sensitivity to statin therapy, leading to improved clinical outcomes. This highlights the clinical significance of prioritizing intensive statin therapy for patients without prior statin use in post‐IVT treatment.

Although statin therapy after IVT can improve clinical outcomes in patients with cardioembolic stroke [26], intensive statin therapy does not seem to enhance this effect. A previous study indicated that in patients with recent stroke or transient ischemic attack without known coronary heart disease, daily administration of 80 mg of atorvastatin slightly increased the incidence of hemorrhagic stroke while still reducing the overall incidence of strokes and cardiovascular events [4]. In our study, we observed that for patients who experienced hemorrhagic transformation, intensive statin therapy did not yield greater benefits compared to non‐intensive therapy. However, it's important to note that in our database, statin administration occurred within 48 h poststroke onset, and some patients received statins after hemorrhagic transformation had already occurred. Therefore, we cannot establish a causal relationship regarding the timing of statin use and its impact on hemorrhagic events.

This study has some limitations. First, as a prospective registry study without randomization, there is inherent selection bias between the intensive and non‐intensive statin groups. Although PSM was used to minimize baseline differences, some confounding factors might have been overlooked, such as the impact of patients taking other statin regimens on clinical outcomes. However, due to regional prescription habits, post‐reperfusion therapy in our database predominantly involved atorvastatin or rosuvastatin. Second, patients not receiving statins were excluded from the analysis due to potential biases from contraindications or delayed use. Considering these patients have a substantial bias compared to those who received statins, which might affect statistical analysis. Similarly, we did not impose a limit on the admission NIHSS score, which may have contributed to some heterogeneity in treatment effects, although this was partially addressed by PSM in the analysis. Third, some potentially relevant data are incomplete, such as data on vessel recanalization and tissue reperfusion before and after IVT. Additionally, the duration of intensive statin therapy mainly occurred during hospitalization, which varied among patients. We also did not include lipid‐lowering effects or adverse events (e.g., liver function impairment and rhabdomyolysis) since these outcomes were not routinely collected in our database. Fourth, the roles of other LDL‐lowering drugs, including ezetimibe, bile acid sequestrants, and PCSK9 inhibitors, in this context remain unclear and require further investigation.

## 5 Conclusion

In the real world, nearly half of the AIS patients receiving IVT also receive combined therapy with intensive statin treatment. Early intensive statin therapy after IVT results in a significant but modest improvement in neurological outcomes, particularly in patients using atorvastatin as the intensive treatment regimen.

## Conflicts of Interest

The authors declare no conflicts of interest.

## Supporting information


Data S1.

## Data Availability

The data that support the findings of this study are available on request from the corresponding author. The data are not publicly available due to privacy or ethical restrictions.
